# Epigenetic Silencing of miR-218-5p Modulates BIRC5 and DDX21 Expression to Promote Colorectal Cancer Progression

**DOI:** 10.3390/ijms26094146

**Published:** 2025-04-27

**Authors:** Hibah Shaath, Radhakrishnan Vishnubalaji, Khalid Ouararhni, Nehad M. Alajez

**Affiliations:** 1 Translational Oncology Research Center (TORC), Qatar Biomedical Research Institute (QBRI), Hamad Bin Khalifa University (HBKU), Qatar Foundation (QF), Doha P.O. Box 34110, Qatar; hshaath@hbku.edu.qa (H.S.); vbradhakrishnan@hbku.edu.qa (R.V.); 2 Genomics Core Facility, Hamad Bin Khalifa University, Qatar Foundation (QF), Doha P.O. Box 34110, Qatar; kouararhni@hbku.edu.qa; 3 College of Health & Life Sciences, Hamad Bin Khalifa University (HBKU), Qatar Foundation (QF), Doha P.O. Box 34110, Qatar

**Keywords:** miRNA, CRC, cancer hallmarks

## Abstract

Colorectal cancer remains one of the leading causes of cancer-related deaths globally. Non-protein coding RNAs, including microRNAs, have emerged as crucial regulators in cancer progression. Herein, we analyzed publicly available datasets for miRNA expression in healthy controls, adenomatous polyps, and colorectal cancer and identified their regulatory networks using HCT116 and HT-29 CRC models. Differentially expressed miRNAs in adenomatous polyps and colorectal cancer were identified, highlighting their role in colorectal cancer initiation and progression. Notably, miR-218-5p was significantly downregulated in adenomatous polyps and colorectal cancer, suggesting a role in colorectal cancer initiation. Functional investigations revealed a tumor suppressive role for miR-218-5p in HCT116 and HT-29 CRC cell models, affecting cell proliferation and three-dimensional organoid formation and promoting cell death. RNA-Seq and bioinformatics identified *BIRC5* and *DDX21* as bona fide gene targets for miR-218-5p, validated by reverse transcription quantitative PCR and Western blotting. Further investigation into the genomic location of *miR-218-5p*, embedded within the *SLIT2* and *SLIT3* introns on chromosome 4 and chromosome 5, respectively, revealed epigenetic silencing through promoter hypermethylation in colorectal cancer cell models. These findings highlight epigenetic silencing of miR-218-5p in colorectal cancer, suggesting its potential as a biomarker and therapeutic target for early detection and intervention.

## 1. Introduction

Despite significant progress in the management of colorectal cancer (CRC), it remains one of the top three causes of cancer-related deaths in both men and women globally, contributing to the overall rise in cancer-related burden [1,2]. The recent research endeavors aim to enhance our comprehension of the molecular changes that lead to CRC development and progression, thus offering potential novel therapeutic opportunities for CRC patients [3,4,5,6].

While protein-coding genes are well recognized for their critical role in CRC, the functions of non-coding RNAs (ncRNAs) in CRC are still being uncovered. Among these ncRNAs, microRNAs (miRNAs) play essential roles in various cellular processes under both normal and disease conditions, including cancer [7]. MiRNAs regulate their biological function via various mechanisms including RNA degradation, protein translation inhibition, acting as toll-like receptor (TLR) ligands, or encoding regulatory peptides [8]. MiRNAs are involved in the regulation of gene expression, maintenance of cellular structure, and participation in various cellular functions [9,10,11] and can function as both tumor suppressors and oncogenes (oncomiRs) depending on their target genes and the cellular context [12]. Tumor-suppressive miRNAs prevent cancer development by inhibiting oncogene expression and regulating pathways involved in cell proliferation, apoptosis, and differentiation, whereas when these miRNAs are downregulated in cancer, it can give way for the overexpression of oncogenic proteins, promoting tumor growth and progression. Oncogenic miRNAs act as oncogenes by suppressing tumor suppressor genes, leading to increased cancer cell survival, proliferation, and metastasis. These upregulated miRNAs in cancer can drive tumorigenesis [13].

The dysregulation of miRNAs and their associated proteins in CRC can have profound effects on key cellular pathways, contributing to tumor initiation, progression, and metastasis. For example, multiple studies have implicated miR-21 [14,15], miR-34a [16,17], miR-143 and miR-145 [18], miR-17-92 cluster [19], miR-31 [20], miR-200 family [21], and miR-223 [22] to have close association with CRC. Regulating the expression of cancer-associated miRNAs has been crucial in the development, differentiation, and progression of CRC. Epigenetic regulation via hypermethylation of miRNA or miRNA host gene promoter regions can lead to reduced miRNA expression. For instance, miR-34a is frequently downregulated in colon cancers due to promoter hypermethylation, leading to the loss of its tumor suppressor activity [23].

In a previous study, we performed miRNA sequencing comparing CRC tissue samples and their non-tumor adjacent tissues, identifying alterations in miRNA, mRNA, and lncRNA expression [24]. However, the role of miRNAs in CRC initiation remains unknown. In the current study, we conducted differential expression analysis on healthy colon, adenomatous polyps (AP), and CRC. AP and CRC are distinct yet related stages along the colorectal neoplastic continuum. Understanding their differences and relationships is crucial for identifying early events in tumorigenesis and developing effective prevention strategies. Through analysis of gene expression profiles, protein–protein interactions (PPIs), transcription factors (TFs), and gene ontology (GO), a significant number of differentially expressed genes (DEGs) were found that were commonly dysregulated in both colorectal polyps and CRC. The presence of these shared DEGs suggests the existence of overlapping molecular alterations and pathways that may contribute to disease initiation and progression [25], with polyp development potentially serving as principal precursors to CRC [26]. These shared genes may serve as promising biomarkers for early detection, diagnosis, and prognosis and may also provide insights into the molecular transition from benign polyps to malignant CRC [25]. This study identified numerous miRNAs to be associated with AP and CRC progression. Notably, miR-218-5p was severely downregulated in AP, as well as in CRC, and found to regulate CRC via targeting key tumor-promoting genes, including Baculoviral IAP Repeat Containing 5 (BIRC5), encoding Survivin, and DExD-Box Helicase 21 (DDX21). MiR-218 is a small ncRNA that plays a significant role in regulating gene expression across various biological processes. Encoded within the introns of the *Slit Guidance Ligand 2* (*SLIT2*) and *Slit Guidance Ligand 3* (*SLIT3*) genes, *miR-218* is evolutionarily conserved among vertebrates [27]. MiR-218 functions predominantly as a tumor suppressor in various cancers including lung cancer [28], cervical cancer [29], and medulloblastoma [30], as well as showing evidence of involvement in cardiovascular implications [31]. MiR-218 has been identified as playing dual roles, functioning either as a tumor suppressor or as an oncomiR, depending on the cancer type, illustrating the complex and context-dependent roles of miR-218 in cancer [32]. Evidence suggesting miR-218’s oncogenic role can be found in oral cancer [33] and triple-negative breast cancer (TNBC) [34]. Our data suggested suppression of miR-218-5p via *SLIT2* and *SLIT3* promoter hypermethylation promotes CRC development and progression, with potential prognostic and therapeutic potential.

## 2. Results

### 2.1. MiRNA Expression Profiling in Healthy Controls, Adenomatous Polyps Presenting, and CRC

**Figure 1:**
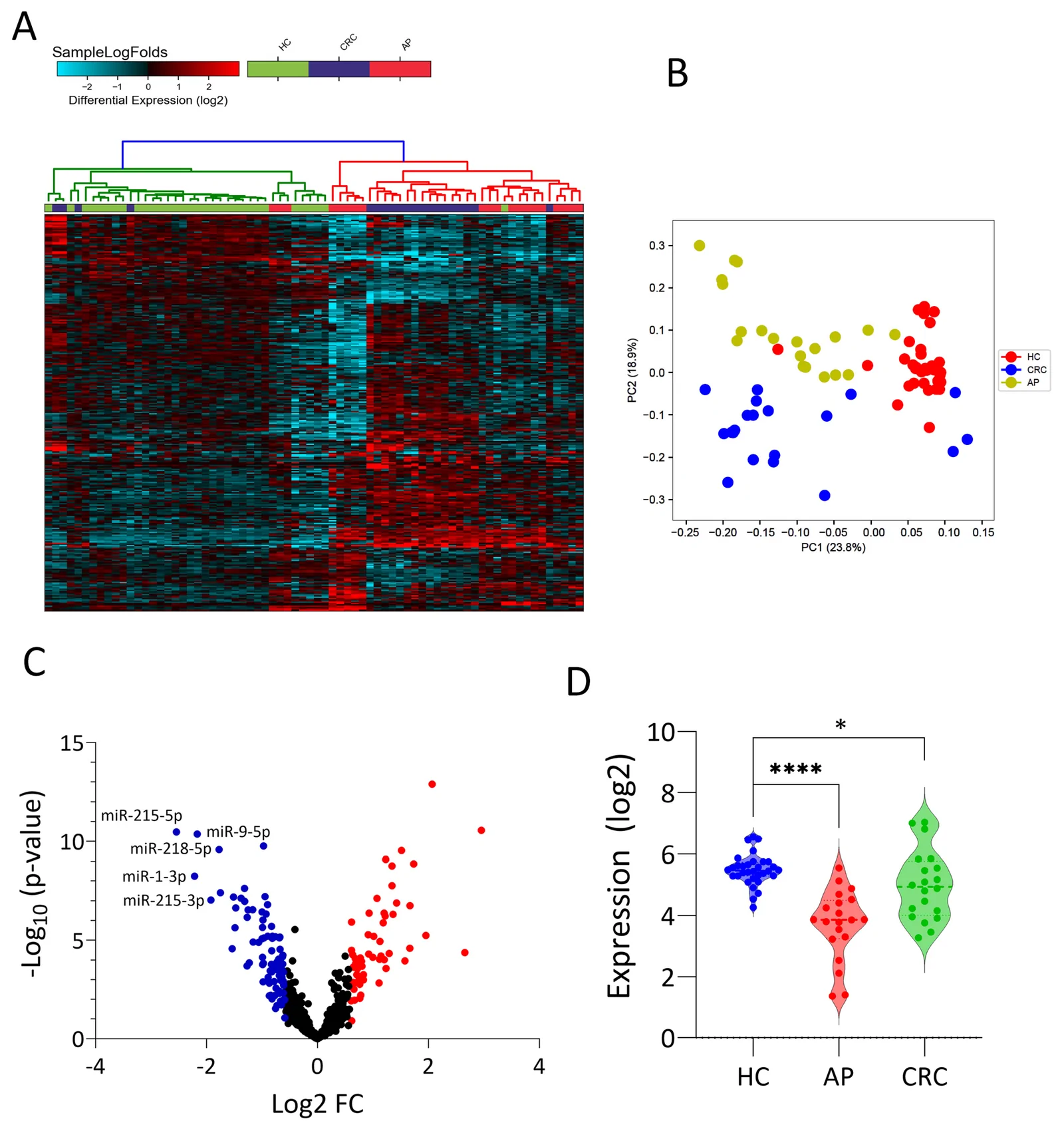
miRNA expression profiling in HC, AP, and CRC. (**A**) Hierarchical clustering of HC (n = 32), AP (n = 20), and CRC (n = 20) based on miRNA expression levels. Each column represents a sample, and each row represents a miRNA transcript. The expression level of each miRNA in a single sample is depicted according to the color scale. (**B**) PCA based on miRNA expression in HC (n = 32), AP (n = 20), and CRC (n = 20). (**C**) Volcano plot depicting the most differentially expressed miRNAs in HC vs. AP, where red indicates upregulated (1.5 FC, *p* < 0.05), blue indicates downregulated (−1.5 FC, *p* < 0.05) expression, and black dots indicate non-significant changes in expression. (**D**) Violin plot comparing the expression of miR-218-5p in HC, AP, and CRC. * *p* < 0.05, **** *p* < 0.00005. HC: healthy control, AP: adenomatous polyp, CRC: colorectal cancer, PCA: principal component analysis. (Data source: PRJNA673192; GEO: GSE160432).

To uncover alterations in miRNA expression during the course of CRC development, we conducted differential miRNA expression analysis on healthy control, AP, and CRC tissues from the PRJNA673192 dataset, employing CLC genomics workbench and the most updated miRBase database. The hierarchical clustering of differentially expressed miRNAs revealed clear segregation among the groups. The majority of samples within the AP group clustered closer to the CRC group, with only a few cases clustering within the healthy group (Figure 1A). These subcategories within the AP group could indicate either a gradual transition from AP to CRC or a distinction between APs that will remain benign, those polyps developing into cancerous lesions. Similar clustering patterns were observed using principal component analysis (PCA) (Figure 1B). Our data identified 55 upregulated miRNAs when comparing AP to healthy controls (HC), 107 upregulated miRNAs when comparing CRC to HC, and 130 upregulated miRNAs when comparing CRC to AP (Table S2). Similarly, we observed 88, 56, and 56 downregulated miRNAs when comparing AP to HC, CRC to HC, and CRC to AP, respectively (Table S3). The Volcano plot depicting differentially expressed miRNAs in AP vs. HC is shown in Figure 1C. The top five downregulated miRNAs in AP vs. HC were miR-215-5p, miR-1-3p, miR-9-5p, miR-215-3p, and miR-218-5p (Figure 1C). Several of the identified miRNAs were previously investigated in the context of CRC [35,36]; however, miR-218-5p was chosen for further investigations based on its documented role in several cancer types and our previous discovery of a tumor suppressive role for this miRNA in nasopharyngeal carcinoma [37]. The expression of *miR-218-5p* in HC, AP, and CRC is illustrated in Figure 1D, depicting significant downregulation of this miRNA, especially during the AP stage (−3.4 FC, *p* = 2.6 × 10^−10^). miR-218-5p was also downregulated but to a lesser extent in CRC compared to HC (−1.4 FC, *p* = 0.03).

### 2.2. miR-218-5p Suppresses Tumorigenicity of CRC Models

**Figure 2:**
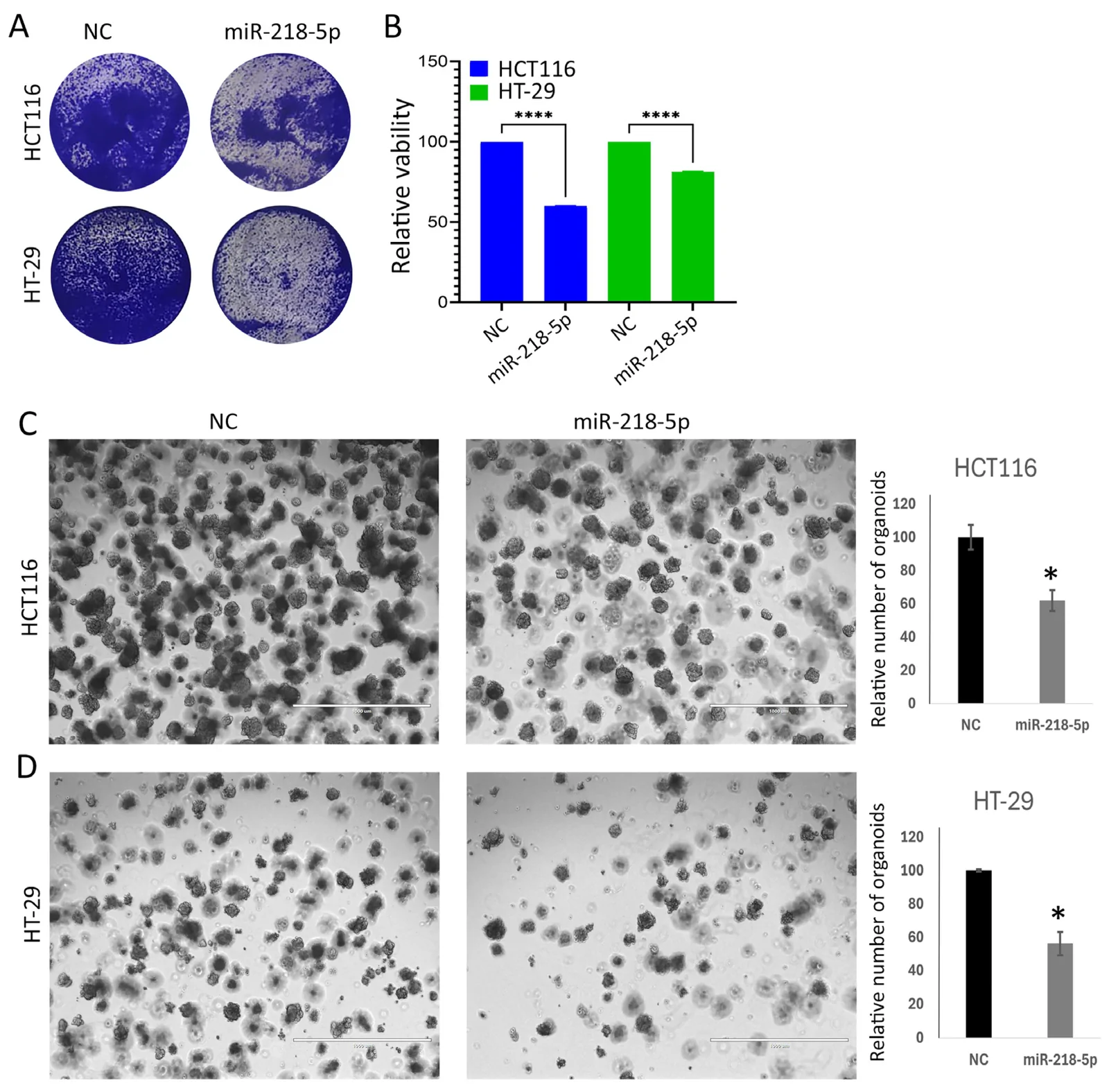
miR-218-5p Suppresses Tumorigenicity of CRC. (**A**) Exogenous expression of miR-218-5p suppresses HCT116 and HT-29 CRC cells’ colony forming unit (CFU) potential. (**B**) Quantification of CFU potential in miR-218-5p mimic and negative control in both cell models. Data are presented as mean ± S.D., n = 2. **** *p* < 0.00005. Representative images illustrating the suppression of HCT116 (**C**) and HT-29 (**D**) growth under 3D conditions in response to exogenous expression of miR-218-5p. (Scale bar = 1000 μM). Quantification data for organoids numbers are presented as relative mean ± S.D., n = 2. * *p* < 0.05.

To assess the biological role for miR-218-5p in CRC, HCT116 and HT-29 were transfected with miR-218-5p mimics, alongside a negative control mimic, and were subsequently assessed for their tumor proliferation potential. To confirm the upregulation of miR-218-5p miRNA after transfection, we performed RT-qPCR using a miScript primer assay and observed remarkable upregulation of miR-218-5p in those cells (Figure S1). Exogenous expression of miR-218-5p inhibited HCT116 and HT-29 cell proliferation compared to control mimic transfected cells (Figure 2A). A quantitative analysis revealed significant suppression (20–40%) of proliferative potential in HCT116 and HT-29 cell models, respectively (Figure 2B). To assess the effects of miR-218-5p overexpression on the ability of CRC cells to form colonies under conditions that recapitulate the three-dimensional (3D) tumor environment, HCT116 and HT-29 were treated with negative control and miR-218-5p mimic and were subsequently grown in Matrigel under 3D culture conditions. The data presented in Figure 2C,D revealed a significant reduction in the number of formed organoids in both cell models in response to miR-218-5p overexpression, thus highlighting a tumor suppressive role for miR-218-5p in CRC under two-dimensional (2D) and 3D culture conditions.

### 2.3. Dead–Live Staining of HCT116 and HT-29 CRC Models in Response to miR-218-5p Overexpression

**Figure 3:**
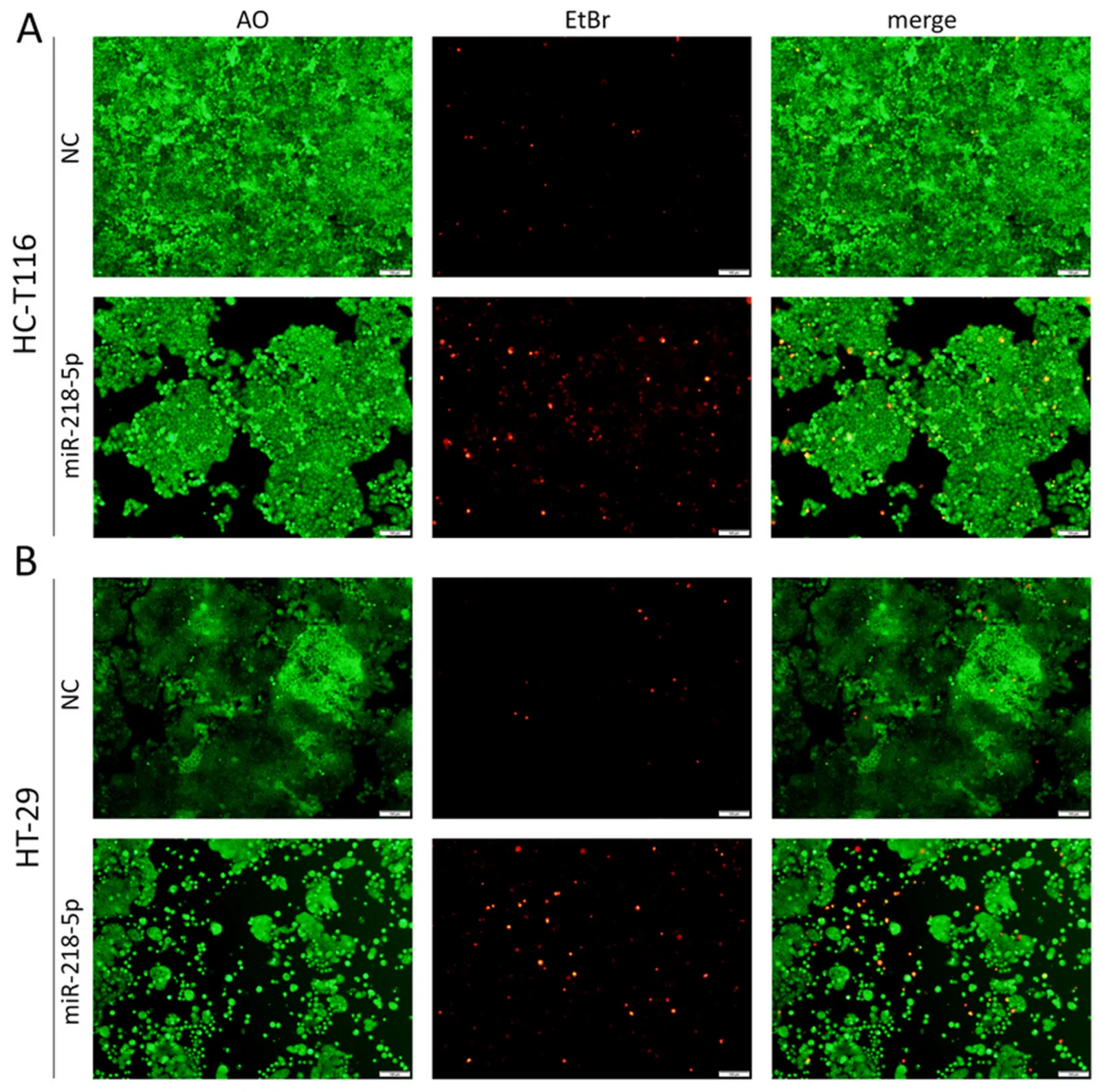
Dead–Live Staining of CRC Models in Response to Exogenous Expression of miR-218-5p. Representative fluorescence images for HCT116 (**A**) and HT-29 CRC (**B**) models post re-expression of miR-218-5p compared to negative control. Cells were stained on day 5 with acridine orange/ethidium bromide to detect live (green) and dead cells (red; necrotic). Scale bar = 100 µm.

To assess the mode of cell death and morphological changes in response to miR-218-5p mimic mediated overexpression, we performed dead–live acridine orange/ethidium bromide (AO/EtBr) staining assay for each condition (Figure 3), which was concordant with our findings and supported our data from the proliferation assay. For both CRC cell models, the non-targeting control produced healthy looking cell morphology with little to no cell death. On the contrary, miR-218-5p overexpression affected cell viability profoundly, where we observed a decrease in the number of cells after 4 days, owing to early cell death and suppressed proliferation. More morphologically condensed looking cells with a substantially higher number of cells stained with EtBr indicating necrotic cells were seen as shown in the second panel and merged images (Figure 3A,B).

### 2.4. Transcriptomic Profiling and Pathway Enrichment Analysis in miR-218 Overexpressing CRC Cells

**Figure 4:**
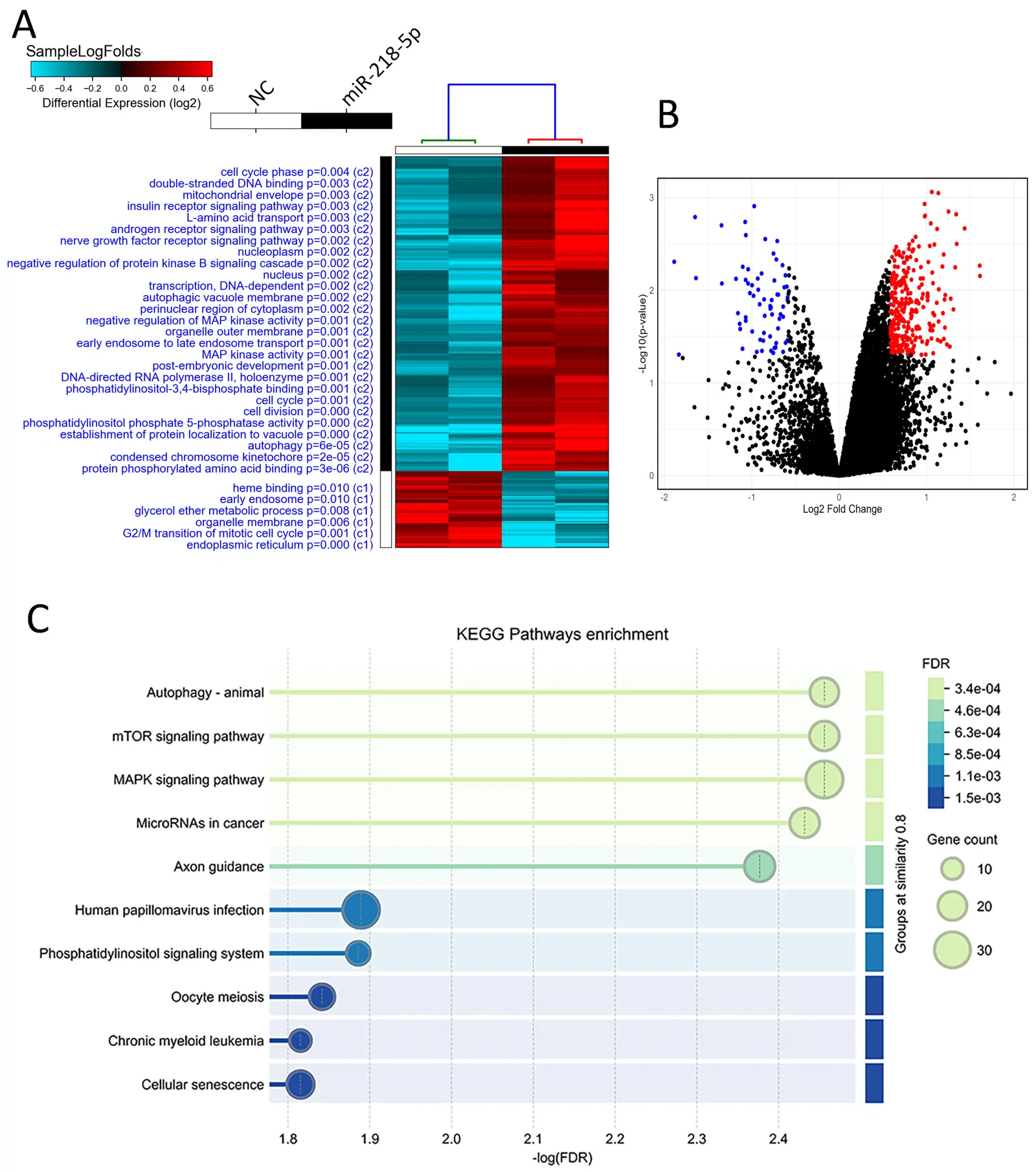
Transcriptomic Profiling of miR-218-5p Overexpressing CRC Cells Revealed a Tumor Suppressor Role for this miRNA. (**A**) Heatmap illustrates alterations in gene expression in HCT116 and HT-29 CRC cells overexpressing miR-218-5p compared to control cells. The expression level for each gene is depicted according to color scale with GO enriched categories and associated *p* values indicated. (**B**) Volcano plot depicting the most differentially expressed genes in miR-218-5p overexpressing compared to control CRC, where red indicates upregulated (>1.5 FC, *p* < 0.05), blue indicates downregulated (<−1.5 FC, *p* < 0.05) expression, and black dots indicate non-significant changes in expression. (**C**) KEGG pathway enrichment analysis based on upregulated genes in miR-218-5p overexpressing CRC cells using STRING protein–protein interactions (PPI) analysis. *X*-axis represents the -log10 false discovery rate (FDR) and *y*-axis represents the enriched category.

To gain more insight into the biological changes in CRC cells due to forced expression of miR-218-5p, CRC cells were harvested on day 3 post transfection, and RNA was extracted. RNA-Seq analysis highlighted clear differential expression patterns segregating miR-218 overexpressing cells from cells transfected with control miRNA mimics (Figure 4A). Interestingly, enriched processes in miR-218 overexpressing CRC cells highlighted autophagy and cell cycle regulation as the most affected functional categories (Figure 4A). Figure 4B shows a volcano plot depicting the most differentially expressed genes in miR-218-5p overexpressing cells compared to control CRC cells, with numerous upregulated genes (1.5 FC, *p* < 0.05) being identified (in red), while blue indicates downregulated genes (−1.5 FC, *p* < 0.05). The list of differentially expressed genes in CRC cells overexpressing miR-218 is provided in Table S4. Kyoto Encyclopedia of Genes and Genomes (KEGG) pathway analysis on the upregulated genes in miR-218 overexpressing HCT116 and HT-29 cells revealed multiple activated pathways, including autophagy and miRNA in cancer (Figure 4C). 

### 2.5. Identification of miR-218-5p Bona Fide Gene Targets

**Figure 5:**
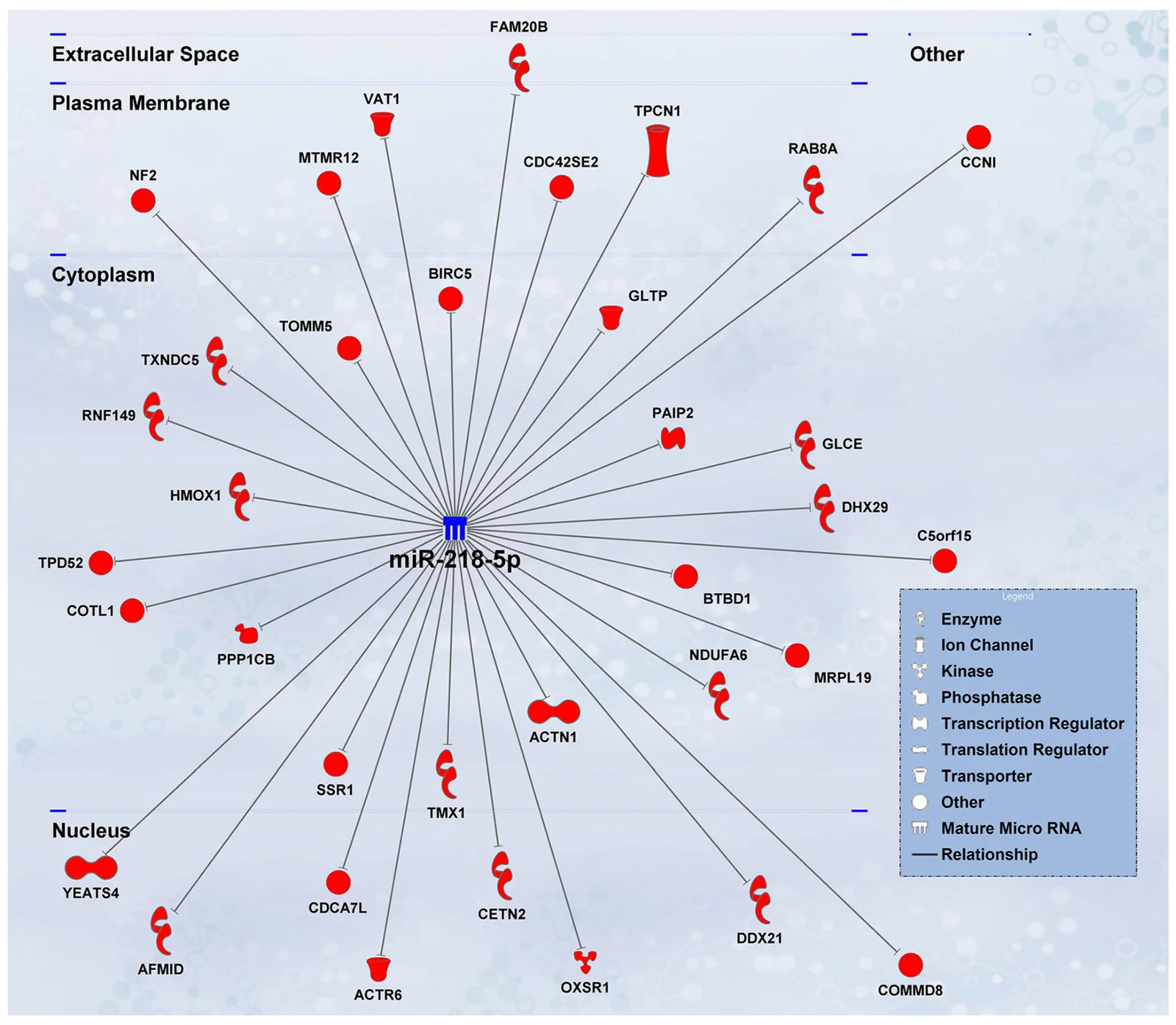
Identification of Bona Fide miR-218-5p Gene Targets. Network illustrating miR-218-5p-mRNA regulatory network in CRC employing the IPA tool. The shape of the symbol indicates the class of each identified gene target according to the figure legend. Blue indicates downregulated expression while red indicates upregulated expression in CRC.

Next, we sought to broaden our insights into finding bona fide mRNA targets for miR-218-5p in CRC cells and identified 41 potential gene targets for miR-218-5p, based on experimental validation and computational predictions (Figure 5 and Table S5). These associations included mRNA targets for ion channels, kinases, phosphatases, transcription and translation regulators, transporters, and others localized in the cell nucleus, cytoplasm, plasma membrane, and extracellular space (Figure 5). Nuclear targets of miR-218-5p include DDX21, Centrin 2 (CETN2), Cell Division Cycle Associated 7 Like (CDCA7L), and Oxidative Stress Responsive Kinase 1 (OXSR1). Many miR-218-5p targets were identified in the cytoplasm including BIRC5, Glycolipid Transfer Protein (GLTP), Poly(A) Binding Protein Interacting Protein 2 (PAIP2), DExH-Box Helicase 29 (DHX29), and Signal Sequence Receptor Subunit 1 (SSR1), along with several others. In the plasma membrane, miR-218-5p was predicted to target Two Pore Segment Channel 1 (TPCN1); RAB8A, member of the RAS oncogene family; Vesicle Amine Transport 1 (VAT1); and Myotubularin-Related Protein 12 (MTMR12). In addition to this, miR-218-5p was projected to target Glycosaminoglycan Xylosylkinase (FAM20B), localized within the extracellular space, as shown in Figure 5.

**Figure 6:**
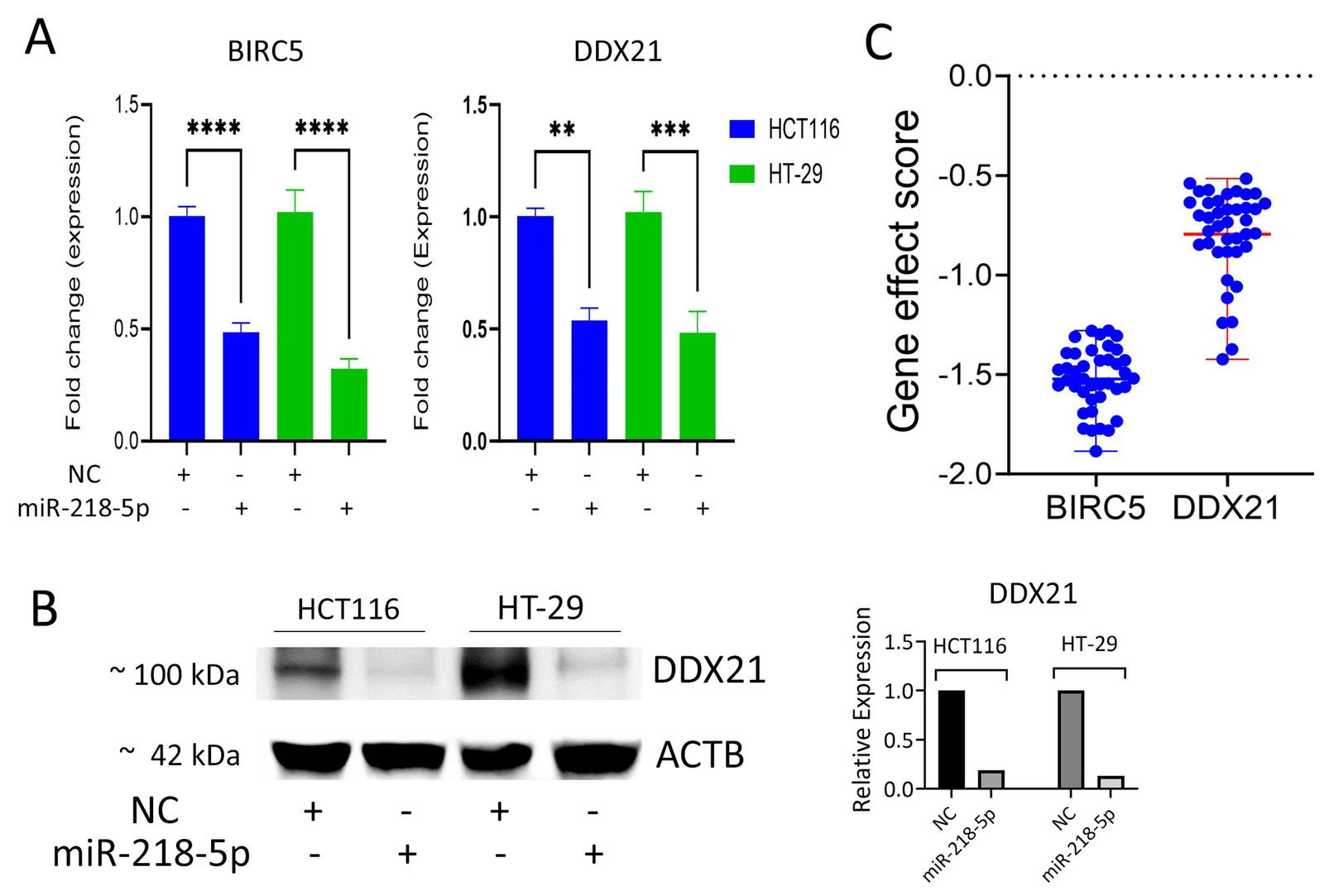
Validation of selected miR-218-5p Gene Targets. (**A**) Validation of BIRC5 and DDX21 as bona fide gene targets for miR-218-5p in CRC. Two-tailed *t*-test was used to compare different groups. ** *p* < 0.005, *** *p* < 0.0005, **** *p* < 0.00005. (**B**) Western blot showing DDX21 protein expression in miR-218-5p overexpressing compared to control HT-29 and HCT116 cells. Quantification of DDX21 protein expression normalized to ACTB is shown in the right panel. (**C**) Gene effect score based on CRISPR-Cas9 screen data in 40 CRC cell models from the DepMap database.

To validate our findings, we further validated two targets, BIRC5 and DDX21, and confirmed their significant downregulation in both HCT116 and HT-29 CRC cell lines upon forced miR-218-5p expression (Figure 6A). Concordant with those data, Western blot analysis revealed substantial reduction in DDX21 protein expression in miR-218-5p transfected HCT116 and HT-29 CRC cells, normalized to ACTB, thus corroborating our findings (Figure 6B). Gene effect scores for DDX21 and BIRC5, derived from CRISPR-Cas9 loss-of-function screens across 40 colorectal cancer (CRC) cell lines in the Achilles project, were −0.8 and −1.5, respectively. In this scoring system, a gene effect score of 0 indicates a non-essential gene, whereas increasingly negative values reflect higher dependency and essentiality for cancer cell survival. A score below −0.5 is typically considered to indicate a gene essential for cell viability. Thus, the scores for *DDX21* and *BIRC5* suggest that both genes are critical for CRC cell survival. These findings support the potential therapeutic relevance of miR-218-5p, which targets DDX21 and BIRC5, as a tumor suppressor in CRC (Figure 6C).

### 2.6. Suppression of miR-218-5p Expression Is Mediated via Promoter Hypermethylation of SLIT2 and SLIT3 Host Genes

**Figure 7:**
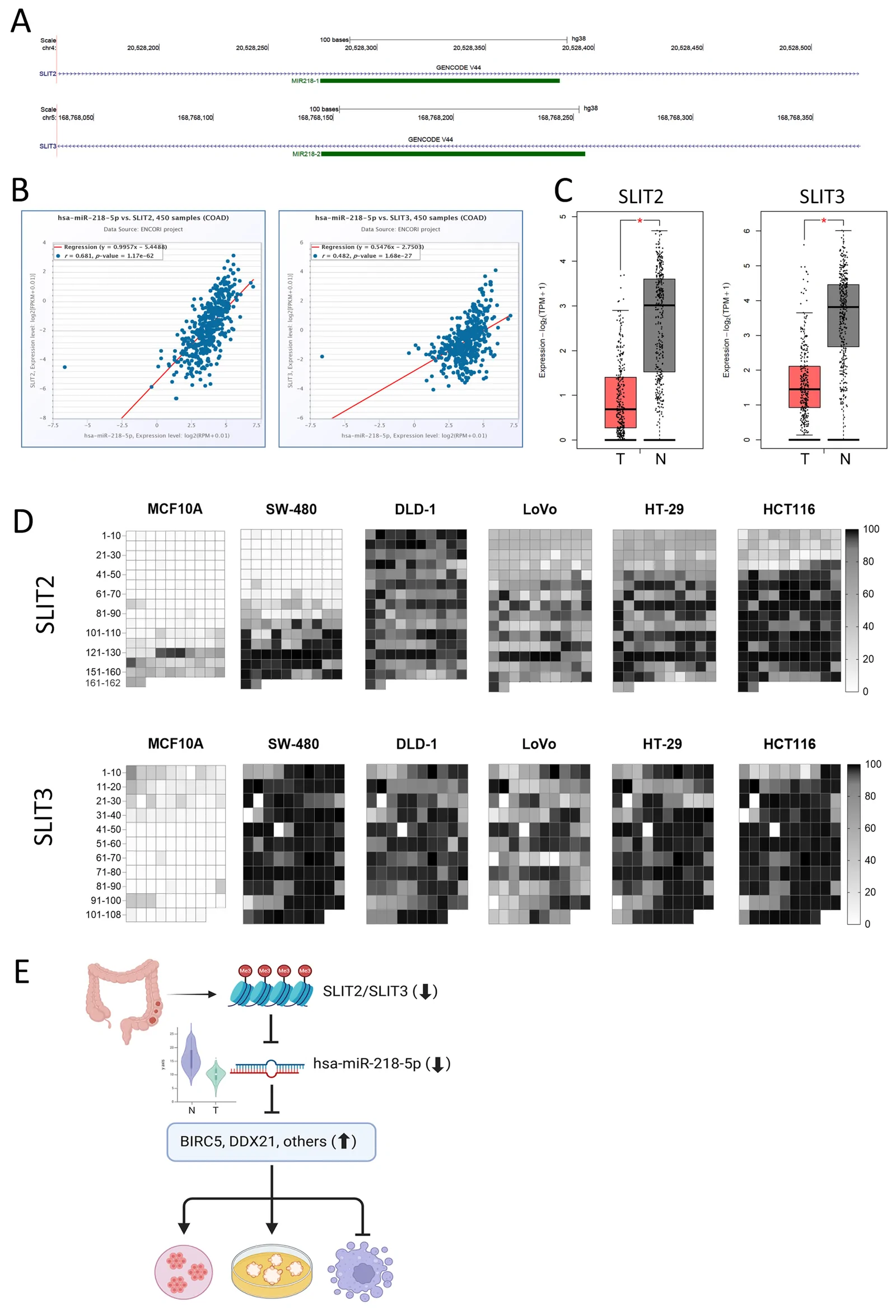
Suppression of *SLIT2* and *SLIT3* miR-218 Host Genes in CRC. (**A**) Genomic location of *miR-218-1* and *miR-218-2* within the *SLIT2* and *SLIT3* genomic region on chromosome 4 and 5, respectively. (**B**) Correlation plot between miR-218-5p and *SLIT2* (**left**) and *SLIT3* (**right**) in a large cohort of COAD (n = 450) from the ENCORI project. (**C**) Downregulation of *SLIT2* (**left**) and *SLIT3* (**right**) in COAD (n = 275) compared to normal colon tissue (n = 349) from GEPIA2 database. T: tumor, N: normal. * *p* < 0.05. (**D**) Methylation analysis of *SLIT2* and *SLIT3* promoters using bisulfite conversion and NGS in a panel of CRC cell models (HCT116, HT-29, SW-480, LoVo, and DLD-1) compared to the MCF10A normal epithelial cells. (**E**) Schematic representation illustrating (🠯) downregulation of *SLIT2* and *SLIT3* in CRC to lead to miR-218-5p suppression (🠯), thus promoting tumorigenesis due to lifted suppression (🠭) of BIRC5, DDX21, and other gene targets identified in the current study.

To gain more insight into the mechanisms leading to miR-218-5p expression in CRC, we observed miR-218-1 and miR-218-2 transcripts to be impeded within the *SLIT2* and *SLIT3* intronic genomic regions on chromosome 4 and 5, respectively (Figure 7A). Concordantly, a strong positive correlation between miR-218-5p and SLIT2/SLIT3 expression was observed in a cohort of 450 colon adenocarcinoma (COAD) from the Encyclopedia of RNA Interactomes (ENCORI) database, suggesting the expression of miR-218-5p is directly correlated with the expression of its *SLIT2* and *SLIT3* host genes (Figure 7B). Data from the Gene Expression Profiling Interactive Analysis (GEPIA2) database showed significant downregulation of *SLIT2* (left) and *SLIT3* (right) expression in a cohort of 275 COAD compared to 349 normal colon tissue samples (Figure 7C). To better understand the mechanism leading to *SLIT2/SLIT3* expression, and subsequently miR-218-5p suppression in CRC, DNA samples from five different CRC cell lines (SW-480, DLD-1, LoVo, HT-29, and HCT116) and one non-cancerous epithelial cell line, MCF10A, were used as input for methylation analysis using the bisulfite method. The distribution of methylation along the reference genomes on chromosomes four (chr4:169834935-169838614) and five (chr5:169836058–169837692) for the promoter regions of *SLIT2* and *SLIT3*, respectively, showed substantial hypermethylation along the entire promoter regions in CRC, in comparison with MCF10A cells (Figure 7D). A notable observation within *SLIT2*, which encodes miR-218-1 within its intronic region, is the detection of higher levels of hypermethylation near the end of the promoter regions in the CRC cell lines, just upstream of the transcription start site, where the TATA box is typically located. However, the promoter region of *SLIT3*, encoding the second miR-218 primary transcript, *miR-218-2*, shows profound hypermethylation across the whole promoter region in CRC compared to normal cells. Taken together, we propose a model where the downregulation of SLIT2 and SLIT3 in CRC via promoter hypermethylation ultimately leads to miR-218-5p suppression, lifting the suppression of downstream targets such as BIRC5 and DDX21, amongst other gene targets identified in the current study, thereby promoting tumorigenesis (Figure 7E).

## 3. Discussion

Exploring novel pathways by expanding our understanding of the functions of ncRNAs, including miRNAs, in the etiology and progression of CRC holds significant promise for enhancing the clinical management of CRC patients. Recent data, including findings from our study, have reinforced the crucial role of miRNAs and their resultant interactions within networks and axes due to their abnormal expression in CRC. Employing transcriptomic profiling and computational analyses, we recently characterized the altered miRNA and mRNA expressions in CRC, compared to normal colon tissue, thus identifying affected signaling pathways, and predicted regulatory networks [24]. In our current study, we investigated the changes in miRNA expression in AP, a condition proceeding CRC, and in CRC compared to normal colon tissue from publicly available miRNA transcriptomic data. Findings from the current study are concordant with Lukosevicius et al., who reported upregulation of miR-1246 and downregulation of miR-215-5p during CRC progression employing the same dataset [38]. Additionally, we identified changes in miRNA expression at the AP stage, including significant suppression of miR-218-5p expression, which was also downregulated in CRC tissues. The tumor suppressor role for miR-218-5p in CRC was validated using proliferation assays under 2D and 3D settings, live–dead staining, and transcriptomic and gene set enrichment analysis in CRC cell models with exogenous miR-218-5p overexpression.

Integration of these data with the IPA miRNA target filter module identified multiple bone fide targets for miR-218-5p with functions spanning various cellular compartments including the nucleus, cytoplasm, plasma membrane, and extracellular space. Among those, survivin (BIRC5), which we were the first to report as a target for miR-218-5p in nasopharyngeal carcinoma (NPC) [37], was identified as a target for miR-218-5p in CRC. Alajez et al. used an integrated tri-modality approach to identify targets for miR-218 in NPC, who subsequently validated direct integrations between miR-218 and the 3′-untranslated regions (UTR) of mRNAs encoding Roundabout Guidance Receptor 1 (ROBO1), survivin (BIRC5), and connexin43 (GJA1) using luciferase-based transcription reporter assays [37]. In laryngeal squamous cell carcinoma, a bioinformatics analysis showed high levels of hsa_circ_0023305 expression in lung squamous cell carcinoma (LSCC) tissue that positively correlated with TNM stage and poorer patient outcomes. hsa_circ_0023305 knockdown inhibited cell proliferation, invasion, and migration by functioning as a miR-218-5p sponge, targeting the melastatin-related transient receptor potential 7 (TRPM7) in LSCC cells. In concordance with what we found in CRC in vitro, miR-218-5p upregulation inhibited LSCC proliferation and invasion both in vivo and in vitro [39].

Due to the genomic positioning of the miR-218 gene in the intronic regions of SLIT2 and SLIT3 [37], in addition to SLIT2 and SLIT3’s reported roles in other cancers [40,41], DNA methylation of promoter regions of both genes was identified as a mechanism leading to epigenetic silencing of miR-218 expression in NPC [37]. *SLIT2*-embedded genes, *SLIT2*-*IT1* and *miR-218*, were downregulated in AML patients. Functionally, the overexpression of SLIT2-IT1/miR-218 demonstrated anti-leukemic effects, impacting cell proliferation, apoptosis, and colony formation both in vitro and in vivo [42,43].

Studies reporting miR-218-5p involvement in CRC are limited; however, one study described a negative role for miR-218-5p in regulating the oncogene Diphthamide biosynthesis 1 (DPH1), with miR-218-5p downregulation driving DPH1’s oncogenic function [44]. This is in concordance with our findings, which showed the upregulation of miR-218-5p to suppress tumor formation potential in CRC. In another study, miR-218-5p overexpression in SW1417 human CRC cells inhibited cell viability and induced cell apoptosis, in alignment with our findings in HCT116 and HT-29. Furthermore, CRC cells with induced high miR-218-5p expression exhibited a downregulation in cellular Fas-associated death domain-like interleukin-1β-converting enzyme inhibitory protein (c-FLIP), insinuating c-FLIP as a direct target of miR-218-5p [45]. In a recent study, TCGA-COAD data and q-PCR validated Ferritin heavy chain 1 pseudogene 3 (FTH1P3) as highly expressed in CRC, promoting migration and invasion of CRC cells by regulating the epithelial–mesenchymal transition (EMT). FTH1P3, directly targeted by Snail Family Transcriptional Repressor 2 (SNAI2), is a transcription factor that enhances its expression. Both FTH1P3 and SNAI2 are repressed by miR-218-5p, which also forms a positive feedback loop with SNAI2 and FTH1P3. This loop plays a critical role in regulating CRC metastasis [46]. The THUMP Domain 3 TRNA Guanosine Methyltransferase (THUMPD3)-AS1/miR-218-5p/(Src Kinase Associated Phosphoprotein 1) SKAP1 axis, reported by Pu et al., promotes CRC cell growth and aggressiveness via THUMPD3-AS1, acting as a competing endogenous RNA (ceRNA) to sponge miR-218-5p and subsequently upregulate SKAP1 expression in CRC cells [47]. Based on our data and other reports in the literature, miR-218-5p has shown to be a versatile player, acting on several fronts involving numerous axes in the progression of CRC. One notable interaction discussed in this paper is the miR-218/DDX21 interaction. Furthermore, the interaction between miR-218 and DDX21 in the cell nucleus was particularly intriguing. DEAD box proteins, including DDX21, are characterized by the conserved Asp-Glu-Ala-Asp (DEAD) motif and are believed to function as RNA helicases. These proteins are involved in various cellular processes that require alterations in RNA secondary structure, such as translation initiation, nuclear and mitochondrial splicing, and the assembly of ribosomes and spliceosomes. DDX21 specifically contributes to unwinding double-stranded RNA, folding single-stranded RNA, and is likely to play critical roles in ribosomal RNA biogenesis, RNA editing, RNA transport, and transcription regulation [48]. Zhang et al. reported DDX21 to be highly expressed in breast cancer and to promote tumorigenesis by enhancing AP-1 activity and rRNA processing. Its expression correlates with cell proliferation rendering DDX21 as a potential therapeutic target in breast cancer [49,50]. In our study, we found miR-218 overexpression in CRC cells to inhibit DDX21 protein expression in HT-29 and HCT116 cell lines; therefore, the loss of miR-218 in CRC could promote CRC progression via the upregulated expression of DDX21.

Further insight into the expression patterns of miR-218-5p identified the miR-218-1 and miR-218-2 transcripts within the *SLIT2* and *SLIT3* genomic regions on chromosomes 4 and 5, respectively. The analysis of 450 COAD samples from the ENCORI project revealed a strong correlation and high significance between the expression of SLIT2 and SLIT3 and that of miR-218-5p. This finding suggests a pathway wherein the downregulation of *SLIT2* and *SLIT3* in CRC ultimately leads to the suppression of miR-218-5p, located within the intronic regions of these genes. SLIT proteins are highly conserved secreted glycoproteins that serve as the primary ligands for ROBOs. The SLIT/ROBO pathway plays a crucial role in various cell signaling processes, including axon guidance, cell migration, motility, and angiogenesis [51]. In the context of cancer, the role of SLIT proteins (particularly SLIT2) is predominantly tumor suppressive. Frequent promoter hypermethylation and transcriptional silencing of SLIT genes in various human cancers, including colorectal, and cervical carcinomas [41,52], suggest a tumor-suppressive function. SLIT2 has been shown to inhibit EMT, suppress angiogenesis, and negatively regulate cell migration and invasion, suggesting a protective role against tumor progression. Promoter hypermethylation of *SLIT2* was identified in 56 out of 66 ovarian cancer samples (84.8%), while reduced *SLIT2* expression was observed in 52 cases (78.8%). A significant association was found between promoter hypermethylation and decreased *SLIT2* expression (*p* < 0.01). Furthermore, the restoration of *SLIT2* expression led to inhibited cell proliferation, migration, and colony formation, along with an increase in apoptosis [53]. Alajez et al. also reported on the tumor suppressive function of miR-218 within a negative feedback loop, where ROBO1, the receptor for SLIT2/3, is targeted by miR-218. This miR-218-ROBO1 circuit plays a critical role in NPC cell migration and brain metastasis [37]. In chronic myeloid leukemia (CML), when patients were categorized by clinical stage, a clear distinction emerged between *SLIT2* promoter hypermethylated and non-hypermethylated groups. The frequency of *SLIT2* hypermethylation increased progressively with disease advancement [42].

SLIT2 is typically a tumor suppressor, where its silencing via promoter hypermethylation in cancer cells is a common mechanism to promote tumor progression by allowing increased migration, invasion, and metastasis [54]. SLIT2 has been shown to inhibit EMT in CRC through the protein kinase B-glycogen synthase kinase 3 β (AKT-GSK3β) signaling pathway [55]; therefore, the hypermethylation of SLIT2 promoter reverses the inhibition of EMT, facilitating tumor progression. In aortic endothelial cells, Slit2 suppresses tumor necrosis factor (TNF)-α-induced vascular endothelial cell proliferation and migration in vitro by inhibiting the vascular endothelial growth factor (VEGF)-Notch signaling pathway [56], meaning the hypermethylation of SLIT2 promoter region and its subsequent suppression means the promotion of TNF-α-induced vascular endothelial cell proliferation and migration, overall abetting tumor progression.

Dickinson et al. focused on the inactivation status of *SLIT3* and *SLIT1* genes in human cancers; *SLIT3* was found to be methylated in 12 out of 29 (41%) of breast cancers, 1 out of 15 (6.7%) lung cancers, 2 out of 6 (33%) colorectal cancers, and in 2 out of 7 (29%) glioma tumor cell lines, while SLIT1 expression shows neuronal specificity [40]. These findings were premises for further investigation into the specific methylation patterns of *SLIT2/3* promoters in CRC cell lines compared to a non-cancerous cell line (MCF10A). While MCF10A cells are widely used as a representative non-cancerous epithelial cell line, they originate from mammary tissue and may not fully recapitulate the characteristics of normal CRC. Future studies utilizing tissue-matched normal CRC cell lines would provide more physiologically relevant comparisons. In our methylation analysis, we revealed significant hypermethylation of the promoter regions for both *SLIT2* and *SLIT3*. Notably, *SLIT3*, whose intron encodes *miR-218-2*, showed prominent methylation, aligning with the suggestion by Kim et al. that the mature form of miR-218 primarily originates from this region [57].

Our study highlights the significant role of miR-218-5p as a potential tumor suppressor in CRC, especially in the early AP stage. Interestingly, miR-218-5p, a known tumor suppressor, was found to be markedly downregulated in AP compared to healthy tissue, whereas its downregulation was less pronounced in CRC relative to healthy controls. This expression pattern suggests that miR-218-5p silencing is an early event in the adenoma–carcinoma sequence. The partial restoration observed in CRC may reflect dynamic changes in regulatory mechanisms during tumor progression, such as compensatory upregulation in response to tumor-associated stress, epigenetic regulation, cellular heterogeneity within CRC samples, or selective pressure favoring cells with intermediate miR-218-5p expression. Despite the relative increase in CRC compared to AP, miR-218-5p remains significantly downregulated in tumors compared to normal tissue, supporting its role as a tumor suppressor and a potential early biomarker in CRC development. Through a combination of RNA-Seq, computational analyses, and functional assays, we have demonstrated the dysregulation of miR-218-5p and its impact on various cellular processes crucial to CRC progression, including cell proliferation, migration, and invasion. Additionally, our findings suggest a complex interaction between miR-218-5p and its target genes, including *BIRC5*, *DDX21*, and various other factors, further underscoring the intricate regulatory networks in CRC. Moreover, the methylation of the *SLIT2* and *SLIT3* genes, which host the miR-218 transcripts, suggests a mechanism by which miR-218-5p expression is suppressed in CRC, promoting tumorigenesis. Taken together, these findings highlight miR-218-5p as a critical regulator of CRC progression, offering promising avenues for early detection and novel therapeutic strategies.

Although we employed a 3D culture system to better mimic the in vivo tumor microenvironment and enhance physiological relevance, the exclusive use of cellular models for validation of the miR-218 overexpression in CRC poses some limitations. While this approach provides controlled conditions for mechanistic insights, it does not fully capture the complexity and heterogeneity of human tissues. Future studies incorporating validation using human colorectal biopsy samples would strengthen the translational relevance of our findings and provide deeper insight into their clinical significance. Despite the promise of miRNAs as a therapeutic strategy for cancer, obstacles such as biodegradation, specificity, stability, and toxicity hinder their wider advancement into clinical trials [58].

Our findings underscore the critical role of non-coding RNAs, particularly miRNAs, in colorectal cancer biology. Specifically, the identification of miR-218-5p as an early and significantly downregulated tumor suppressor during the adenoma-to-carcinoma transition advances our understanding of early CRC development. Moreover, the demonstration of its direct regulation of essential oncogenic targets such as BIRC5 and DDX21 supports its potential as both a diagnostic biomarker and a therapeutic target. Further exploration of miR-218-5p and its regulatory network may yield novel strategies for early detection and targeted intervention in CRC, offering tangible clinical benefits for patients.

## 4. Materials and Methods

### 4.1. miRNA Quantification and Bioinformatics Analysis

Raw miRNA expression data (FASTQ) files including 32 healthy controls, 20 AP, and 20 CRC were retrieved from the PRJNA673192 study using the Sequence Read Archive (SRA) toolkit v2.9.2 (National Center for Biotechnology Information, Bethesda, MD, USA) [59]. FASTQ files were subjected to small-RNA quantification and differential expression analysis in QIAGEN CLC Genomics Workbench 20.2 (QIAGEN, Aarhus, Denmark) using built-in workflow. Initially, the raw expression data were trimmed then mapped to the miRbase v22 reference genome. Read counts of mature miRNA were then normalized using the trimmed mean of M values (TMM) normalization method. Mature miRNA expression values were then subjected to differential analysis using Altanalyze v.2.1.3 [1,60].

### 4.2. Maintenance of Cancer Cell Lines

Human CRC (HCT116 and HT-29 cell lines) were cultured in Dulbecco’s Modified Eagle’s Medium (DMEM) (Gibco, Cat. No. 11965-092, Thermo Fisher Scientific, Waltham, MA, USA). All culture media were supplemented with 10% fetal bovine serum (FBS) (Cat. No. 16000-044, Thermo Scientific, Rockford, IL, USA) and 1% penicillin/streptomycin (Cat. No. 15140-122, Thermo Scientific, Rockford, IL, USA). Cells were cultured as an adherent monolayer at 37 °C under 5% CO_2_ in a humidified incubator.

### 4.3. mirVana miRNA Mimic Transfection in CRC Cells

To investigate the functional role of miR-218-5p in regulating CRC biology, HCT116 and HT-29 cell lines were transfected with hsa-miR-218-5p mirVana miRNA mimics (Cat. No. 4464066) and negative control, mirVana miRNA mimic negative control #1, Cat. No. 4464058), Ambion (Thermo Fisher Scientific, Waltham, MA, USA). In brief, miRNA mimic (at a final concentration of 30 nM) was diluted in Opti-MEM (Cat. No. S11058-021; Gibco, Thermo Fisher Scientific, Carlsbad, CA, USA), and 1.5 μL of Lipofectamine™ 2000 (Cat. No. 52758; Invitrogen, Waltham, MA, USA) was diluted in 50 μL Opti-MEM. After 20 min incubation at ambient temperature, 200 μL of transfection mixture was added to 800 μL of transfection medium containing 0.168 × 10^6^ cells/mL, and the wells were then topped up with extra medium after 24 h.

### 4.4. Cell Proliferation Assay

The proliferative ability of HCT116 and HT-29 cells transfected with miR-218-5p mimics or negative control was determined using clonogenic assay and Crystal Violet (ACS reagent, ≥90.0% anhydrous basis; Sigma-Aldrich, Merck KGaA, Darmstadt, Germany). The transfected cells were seeded in 12-well plates, and on day 5 post treatment, the wells were washed and stained. Once dry, the plates were scanned, and the number of colonies was observed under an inverted microscope. To quantify the data, the wells were covered with 10% sodium dodecyl sulfate (SDS) solution (Sigma-Aldrich, Cat. No. L3771, St. Louis, MO, USA) and were left on a rocker to dissolve the crystal violet. Once completely dissolved, 200 μL of the solution was transferred to a 96-well plate (in duplicates) and checked for absorbance using a plate reader NanoQuant Plate and Infinite M200 Pro reader (Tecan, Männedorf, Switzerland). 

### 4.5. Detection of Cell Death Using Fluorescence Microscopy

The AO/EtBr fluorescence staining method was used to assess apoptosis/necrosis in treated CRC cells vs. control. In brief, CRC cells under various experimental conditions and cultured in a 12-well flat-bottom tissue culture plate were washed twice with phosphate buffered saline (PBS) (Gibco, Thermo Fisher Scientific, Cat. No. 10010-023, Waltham, MA, USA) on day 4 and subsequently were stained with dual fluorescent staining solution containing 100 mg/mL AO and 100 mg/mL EtBr (AO/EtBr, Sigma Aldrich, St. Louis, MO, USA) for 2 min. Subsequently, the cells were observed and imaged under an Olympus IX73 fluorescence microscope (Olympus, Tokyo, Japan). The AO/EtBr staining method was employed to identify apoptotic cells, with AO used to visualize nuclear shape and potential apoptosis, while EtBr-positive cells (red) indicated necrotic cells.

### 4.6. 3D Organoid Culture

The cells were harvested on day 4 post transfection with miR-218-5p mimics or control and were suspended in Corning Matrigel^®^ Matrix^®^ (Corning, Cat. No. 354234, Corning, NY, USA). Using 6 cm dishes, matrigel-containing cells were pipetted into the center of the dish forming 3 separate cell containing domes. Dishes were placed in a humidified incubator to facilitate polymerization of the Matrigel^®^ (37 °C, 20 min) to solidify the domes and growth medium (DMEM) was then added to the dish, covering the domes. Organoid formation and growth were monitored periodically, and images were taken on day 7 using the EVOS™ Cell Imaging System (Thermo Fisher Scientific, Waltham, MA, USA). Quantification of the numbers of organoids was conducted using OpenCFU software, version 3.9.0, as described before [61].

### 4.7. Total RNA Isolation and Next-Generation Sequencing (NGS) in miR-218-5p Overexpressing CRC Cells

To identify potential gene targets for miR-218-5p, HCT116 and HT-29 CRC cells were transfected with miR-218-5p mimic or negative control. At 72 h, total RNA was extracted from transfected cells, and subsequently, library preparation was carried out using the TruSeq Stranded Total RNA Library (Illumina Inc., San Diego, CA, USA), followed by NGS using the Illumina NextSeq 2000 at approximately 50 million paired end reads (2 × 75 bp) per sample, as we described before [11]. The generated FASTQ files were subsequently aligned and mapped to the hg38 reference genome using the CLC Genomics Workbench v21.0.5. Normalized transcript per million (TPM) expression values were subsequently subjected to differential expression analysis using 2.0-fold change and <0.05 false discovery rate (FDR)-adjusted p-value cut-off in Altanalyze v.2.1.3. KEGG pathway analyses were conducted using STRING version 12.0 database [62].

### 4.8. Identification of miR-218-5p Targets

The miRNA Target Filter in IPA (using the default database) was subsequently employed to identify miRNA–mRNA networks based on differentially expressed mRNAs in miR-218-5p overexpressing CRC cells. Data illustrating the interactions between miR-218-5p and potential gene targets was found using the Path Designer tool in IPA. The gene effect score was retrieved from the genome-wide CRISPR-Cas9 functional screen data from the Achilles project in CRC models as described before [63,64]. Correlations between miR-218 and BIRC5 or DDX21 and their expressions in CRC compared to normal colon tissue were retrieved from the ENCORI and GEPIA2 databases, respectively [65,66].

### 4.9. Reverse Transcriptase Quantitative Polymerase Chain Reaction (RT-qPCR)

RT-qPCR was used to validate the expression of BIRC5 and DDX21 in CRC cells post miR-218-5p mimic transfection compared to negative control transfected cells. RNA extracted from the cells (500 ng) was reverse transcribed to cDNA using the High-Capacity cDNA Reverse Transcription kit (Thermo Fisher Scientific, Waltham, MA, USA). Subsequently, qPCR was performed using specific primer pairs as detailed in Table S1 and the PowerUp SYBR Green Master Mix (Thermo Fisher Scientific, Waltham, MA, USA) on the QuantStudio™ 6 Flex Real-Time PCR System (Applied Biosystems, Thermo Fisher Scientific, Waltham, MA, USA). The mRNA transcript levels of the target genes were determined based on their respective CT values, normalized against β-actin (ACTB) transcript levels, and presented as FC using the 2^−ΔΔCt^ method compared to control cells.

### 4.10. Western Blot

The cells were harvested on day 4 post transfection with the indicated control and miR-218 mimics and collected in a radio-immunoprecipitation assay (RIPA) lysis and extraction buffer (Thermo Fisher, Cat. 89900) with Halt™ Protease Inhibitor Cocktail (Cat. 78438). Protein from each sample was quantified using Pierce™ BCA Protein Assay Kit (Cat. No. 23225; Thermo Fisher Scientific, Waltham, MA, USA) and read using a FLUOstar Omega Microplate Reader (BMG LABTECH, Ortenberg, Germany), compared to albumin standards. Then, 30 μg of protein was combined with 2× Laemmli Sample Buffer (Bio-Rad, Cat. No. 161-0737, Hercules, CA, USA)and denatured at 95 °C for 5 min. Samples were loaded in 10% Mini-PROTEAN^®^ TGX™ Precast Gels (Bio-Rad Laboratories, Cat. No. 4561034, Hercules, CA, USA) with PageRuler™ Plus Prestained Protein Ladder (Thermo Scientific, Cat. No. 26619, Waltham, MA, USA) in running buffer (10× Tris/Glycine/SDS Buffer: Bio-rad, Cat. 1610732). After the run, the gel was assembled utilizing the Trans-Blot Turbo Mini 0.2 µm PVDF Transfer Packs (Bio-Rad, Cat. No. 1704156, Hercules, CA, USA) and put into the Trans-Blot Turbo Transfer System (Bio-Rad, Hercules, CA, USA) for semi dry blotting for 3 min. The membrane was immersed in 5% blotting-grade blocker non-fat dry milk (Bio-Rad Cat. 1706404XTU) in TBST (10× TBS: (Bio-Rad, Cat. 1706435, Tween 20: Bio-Rad, Cat. 1706531). After 1 h, primary DDX21 Antibody (D-8) (Santa Cruz Biotechnology, Cat. No. sc-376953, Dallas, TX, USA) was diluted in TBST 1:250 and incubated overnight at 4 °C on a rocker and then for 1 h in anti-mouse IgG, HRP-linked Antibody 1:2000 (Cell Signaling Technology, Cat. No. 7076, Danvers, MA, USA) the next day. ACTB was detected on the same blot (β-Actin Rabbit: Cell signaling mAb, Cat. 4970. Anti-rabbit IgG, HRP-linked Antibody, 1:2000: Cell signaling, Cat. 7074). Blots were visualized on the Bio-Rad imager and bands were quantified Image Lab Software, Version 6.1.0, Build 7, Standard Edition (Bio-Rad Laboratories Inc., Hercules, CA, USA).

### 4.11. Methylation Analysis

Genomic DNA extracted from SW480, DLD-1, LoVo, HT-29, and HCT116 CRC cells compared to MCF10A normal epithelial cells and treated with Proteinase K was used as input for methylation analysis by the bisulfite method using the EZ DNA Methylation Kit (Zymo Research, Cat. No. D5001, Irvine, CA, USA) according to the manufacturer’s protocol. The DNA input was 400 ng. After bisulfite conversion, the DNA was subjected to PCR amplification using a specific set of primers (Table S1) to amplify CpG islands of SLIT2 and SLIT3 genes using HotStar DNA polymerase enzyme from Qiagen (Cat. No. 203205, Hilden, Germany). We used a set of new primers designed using the Bisulfite Primer Seeker tool (https://zymoresearch.eu/pages/bisulfite-primer-seeker?srsltid=AfmBOorU4eOvNH-8wO0N7uw0Kwx1fChVU475tZCtUxBHWRMsoYlJM7Ak (accessed on 25 May 2024)), in addition to the previously published primer sequences for the SLIT2 and SLIT3 promoter regions [40,43]. The amplification was performed in a thermal cycler: 95 °C for 15 min, followed by 35 cycles of 95 °C for 1 min, 55 °C for 1 min and 72 °C for 2 min, and then 72 °C for 5 min. The quality of the generated amplicons was checked using the Agilent TapeStation System (Agilent Technologies, Santa Clara, CA, USA) and quantified using Qubit™ Fluorometer (Thermo Fisher Scientific, Waltham, MA, USA). Libraries were prepared using Illumina DNA Prep (Illumina, Cat. No. 20015964, San Diego, CA, USA) according to the manufacturer’s protocol. The quality of the generated libraries was checked on an Agilent 2100 Bioanalyzer system (Agilent Technologies, Santa Clara, CA, USA) and quantified using the Qubit system. Libraries that passed quality control were pooled and sequenced on the MiSeq system (Illumina, San Diego, CA, USA)) with a minimum of 100 thousand paired end reads (2 × 150 bp) per sample.

## Data Availability

Processed data are available in Supplementary Data Files.

## Supplementary Materials

The following supporting information can be downloaded at: https://www.mdpi.com/article/10.3390/ijms26094146/s1.

## Abbreviations

The following abbreviations are used in this manuscript:

CRC—colorectal cancer, ncRNAs—non-coding RNAs, miRNAs—microRNAs, mRNA—messenger RNA, lncRNA—long non-coding RNA, AP—adenomatous polyps, IPA—ingenuity pathway analysis, BIRC5—Baculoviral IAP Repeat Containing 5, DDX21—DEAD-Box Helicase 21, SLIT2—Slit Guidance Ligand 2, SLIT3—Slit Guidance Ligand 3.
