# Supplementary material for: Epigenetic Silencing of miR-218-5p Modulates BIRC5 and DDX21 Expression to Promote Colorectal Cancer Progression
Source: Int J Mol Sci. 2025 Apr 27;26(9):4146. doi: 10.3390/ijms26094146 (PMC12071466; doi:10.3390/ijms26094146)
Supplement: Supplementary file 1 [file ijms-26-04146-s001.zip › Figure S1.pdf]

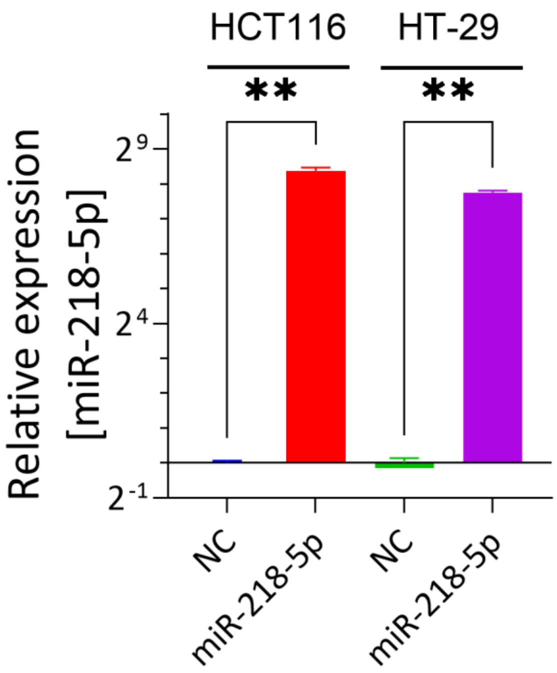

Figure S1. Relative miR-218-5p expression in transfected CRC cells. Representative plot illustrating relative miR-218-5p expression in HCT116 and HT-29 cells on day 3 post transfection. Expression levels were normalized to RNU44 expression. Data are presented as mean ± S.E.M., n=3. ** $p < 0.005$.
