# Supplementary material for: Epigenetic Silencing of miR-218-5p Modulates BIRC5 and DDX21 Expression to Promote Colorectal Cancer Progression
Source: Int J Mol Sci. 2025 Apr 27;26(9):4146. doi: 10.3390/ijms26094146 (PMC12071466; doi:10.3390/ijms26094146)
Supplement: Supplementary file 1 [file ijms-26-04146-s001.zip › Table S1.pdf]

Table S1. SYBR green and methylation primer sequences used in current study.

Names	Primer Sequences (5′-3′)
ACTB_F1	GGCACCCAGCACAATGAAG
ACTB_R1	CCGATCCACACGGAGTACTTG
BIRC5_F1	CAGACTTGGCCCAGTGTTTC
BIRC5_R1	GTTCCTCTATGGGGTCGTCA
DDX21_F1	GTGGAGCAAAAAGCGGGAAT
DDX21_R1	GCACGGAATCCAAAAGCCTG
**Methylation primers**	
SLIT2_F1	GATTTGGTGTTATTTATTTGGGAAG
SLIT2_R1	ACACCTAATAACAAAAATAAAAAAAACA
SLIT2_F2	YGGTTTTTGTTTTTTTTATTTTTGTTA
SLIT2_R2	AAACTCCAAAAAACCAAATAAAAAATAA
SLIT2_F3	TTTATTTTTTATTTGGTTTTTTGGAGTT
SLIT2_R3	CAACRATAATTTACAAACACACATAC
SLIT2_F4	AGTGTTGATTAGTGGATATTTTTGTT
SLIT2_R4	TCTTCTATCTCCCAAAAATAAACTT
SLIT2_F5	TATTTTAGTAGTYGTTGGAAGTTTTAG
SLIT2_R5	ACACTAAAAACTTTCTTTAACTTACTAC
SLIT3_F1	GGTYGTTTTAGTAAAGTTTTTAATTTAG
SLIT3_R1	AAATCCRACTTAAAACTAAACTAAAAAA
SLIT3_F2	GATGGAGTTGTTGTTGTTGTY
SLIT3_R2	CTAACAACRAAACAAATACACTTAA
SLIT3_F3	TTTTTTAGGAGYGGGGTTTTGTATATT
SLIT3_R3	CCCTCRATAAAACCCAAATAAATAA
SLIT3_F4	GGTTAGTTTATTYGGTYGTTTYGTG TTTTAGT
SLIT3_R4	TACCCACCCRAAAACCATAATATACAAAA
